# 3-(4-Chloro­phen­yl)-4-(4-methoxyphen­yl)-6-(phenyl­selenylmeth­yl)-2,3,3a,3b,4,5,5a,6,1′′,2′′,3′′,4′′-do­deca­hydro­azeto[2′,3′:3,4]pyrrolo[1,2-*b*]isoxazole-2-spiro-2′′-naphthalene-5,1′′-dione

**DOI:** 10.1107/S1600536808006570

**Published:** 2008-03-14

**Authors:** E. Theboral Sugi Kamala, S. Nirmala, L. Sudha, N. Arumugam, R. Raghunathan

**Affiliations:** a Department of Physics, Easwari Engineering College, Ramapuram, Chennai 600 089, India; b Department of Physics, SRM University, Ramapuram Campus, Chennai 600 089, India; c Department of Organic Chemistry, University of Madras, Guindy Campus, Chennai 600 025, India

## Abstract

In the title compound, C_36_H_31_ClN_2_O_4_Se, the four-membered β-lactam ring is fused to a pyrrolidine ring. The central five-membered ring of the fused tricyclic system exhibits an envelope conformation with the N atom as the flap, while the other five-membered ring exhibits a twist conformation. The chloro­phenyl ring is almost perpendicular to the pyrrolidine ring, making a dihedral angle of 73.45 (1)°. The crystal structure is stabilized by weak inter­molecular C—H⋯O inter­actions and the packing is further enhanced by C—H ⋯N inter­actions and π–π inter­actions between benzene rings of tetra­lone groups in mol­ecules related by an inversion center, with a centroid–centroid separation of 3.8923 (2) Å.

## Related literature

For related literature, see: Allen *et al.* (1987 ▶); Amal Raj *et al.* (2003 ▶); Brakhage (1998 ▶); Cremer & Pople (1975 ▶); Kilonda *et al.* (1995 ▶); Nardelli (1983 ▶).
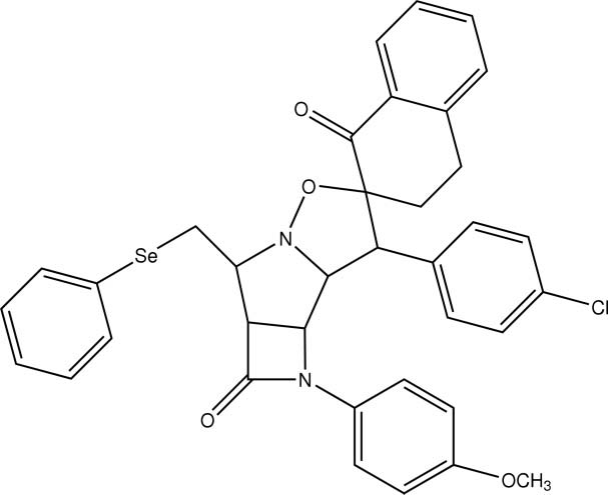


## Experimental

### 

#### Crystal data

C_36_H_31_ClN_2_O_4_Se
*M* *_r_* = 670.04
Monoclinic, 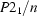

*a* = 14.4697 (3) Å
*b* = 10.9493 (3) Å
*c* = 19.3011 (4) Å
β = 94.661 (1)°
*V* = 3047.82 (12) Å^3^
*Z* = 4
Mo *K*α radiation
μ = 1.36 mm^−1^
*T* = 293 (2) K
0.30 × 0.30 × 0.24 mm

#### Data collection

Bruker APEXII diffractometer
Absorption correction: multi-scan (Blessing, 1995 ▶) *T*_min_ = 0.671, *T*_max_ = 0.720
37171 measured reflections
8699 independent reflections
5453 reflections with *I* > 2σ(*I*)
*R*_int_ = 0.035

#### Refinement

*R*[*F* ^2^ > 2σ(*F* ^2^)] = 0.053
*wR*(*F* ^2^) = 0.176
*S* = 1.02
8699 reflections
398 parameters
H-atom parameters constrained
Δρ_max_ = 0.72 e Å^−3^
Δρ_min_ = −0.86 e Å^−3^

### 

Data collection: *APEX2* (Bruker, 2004 ▶); cell refinement: *SAINT* (Bruker, 2004 ▶); data reduction: *SAINT*; program(s) used to solve structure: *SIR92* (Altomare *et al.*, 1993 ▶); program(s) used to refine structure: *SHELXL97* (Sheldrick, 2008 ▶); molecular graphics: *ORTEP-3* (Farrugia, 1997 ▶); software used to prepare material for publication: *PLATON* (Spek, 2003 ▶).

## Supplementary Material

Crystal structure: contains datablocks I, global. DOI: 10.1107/S1600536808006570/bh2162sup1.cif

Structure factors: contains datablocks I. DOI: 10.1107/S1600536808006570/bh2162Isup2.hkl

Additional supplementary materials:  crystallographic information; 3D view; checkCIF report

## Figures and Tables

**Table 1:** Hydrogen-bond geometry (Å, °)

*D*—H⋯*A*	*D*—H	H⋯*A*	*D*⋯*A*	*D*—H⋯*A*
C4—H4⋯O4^i^	0.98	2.57	3.305 (3)	132
C23—H23⋯O2	0.98	2.32	2.803 (3)	109
C23—H23⋯N1	0.98	2.54	2.918 (3)	103

Symmetry code: (i) 
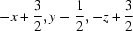
.

**Fig. 1.**
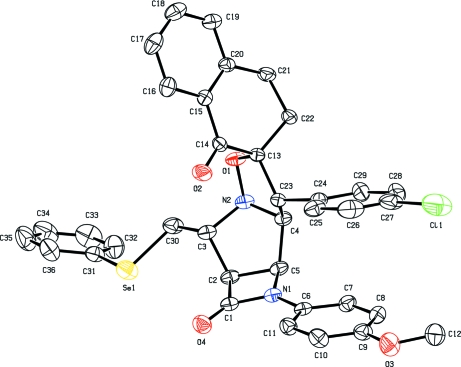
The molecular structure of (I) with 30% probability displacement ellipsoids.

**Fig. 2.**
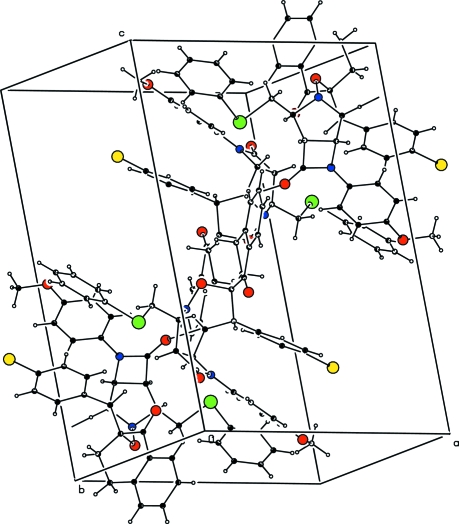
The packing of the molecules viewed along c axis.

## supplementary crystallographic information

### Comment

Pyrrolidines and pyrroles are common structural motifs in drugs and drug candidates owing to their ability to act as selective glycosidase inhibitors, which are used in the treatment of diabetes, cancer, malaria and viral infections, including AIDS (Kilonda *et al.*, 1995). These derivatives also possess antimicrobial and antifungal activity (Amal Raj *et al.*, 2003). The discovery of β-lactam is very significant in the history of therapeutic medicine as an antibiotic in the form of penicillin and cephalosporin for infectious diseases (Brakhage, 1998). Due to their importance, the crystal structure determination of the title compound, (I), was carried out and the results are presented here.

Figure 1 shows the *ORTEP-3* (Farrugia, 1997) plot of compound (I). Bond lengths and angles are comparable with other reported values (Allen *et al.*, 1987).

In the molecule the five membered ring N2/C3/C2/C5/C4 exhibits *envelope* conformation with envelope on N2 and with an asymmetry parameter (Nardelli, 1983) Δ_s_(N2) = 0.0067 (1) and with the puckering parameters (Cremer & Pople, 1975) q2 = 0.3669 (3) Å and φ2 = 70.58 (4)°. Another five membered ring C4/C23/C13/O1/N2 exhibits *twist* conformation with asymmetry parameters Δ_s_(C4) = 0.0297 (13), Δ_2_(O1) = 0.0414 (9) and with the puckering parameters q2 = 0.3452 (2) Å and φ2 = -173.27 (4)°.

The sum of bond angles around atom N1, 359.8°, and around atom N2, 324.57°, indicate *sp*^2^ and *sp*^3^ hybridizations, respectively. The chlorobenzene ring is almost perpendicular to the pyrrolidine ring, making a dihedral angle of 73.45 (1)°. The anisole ring makes a dihedral angle of 14.85 (1)° with the central β-lactam ring, while angle between anisole and tetrahydronapthanone is 66.38 (1)°.

In the crystal packing, atoms O2 and O4 are involved in intermolecular and intramolecular C—H···O interactions, along with C—H···N interactions. Weak intermolecular π···π interactions occur within the benzene ring C15···C20 (1 - *x*, -*y*, 1 - *z*), with a centroid-to-centroid separation of 3.8923 (2) Å.

### Experimental

To a solution of the bicyclic nitrone (1 mmol) in dry acetonitrile (20 ml) was added 4-chlorobenzilidene tetralone (1 mmol) under N_2_ atmosphere. The mixture was refluxed for 4 h. After completion of the reaction, the solvent was distilled off under reduced pressure. The crude product was purified by column chromatography (hexane:ethyl acetate, 8:2) to give pure β-lactam (I) in good yield. The product was recrystallized from dry benzene by slow evaporation.

### Refinement

H atoms were placed in idealized positions and allowed to ride on their parent atoms, with C—H bond lengths fixed to 0.93 (aromatic CH), 0.96 (methyl CH_3_), 0.97 (methylene CH_2_) or 0.98 Å (methine CH), and *U*_iso_(H) = 1.2–1.5*U*_eq_(carrier C).

### Crystal data

**Table:**

C_36_H_31_ClN_2_O_4_Se	*F*_000_ = 1376
*M**_r_* = 670.04	*D*_x_ = 1.460 Mg m^−^^3^
Monoclinic, *P*2_1_/*n*	Mo *K*α radiation λ = 0.71073 Å
Hall symbol: -P 2yn	Cell parameters from 37171 reflections
*a* = 14.4697 (3) Å	θ = 1.7–29.8º
*b* = 10.9493 (3) Å	µ = 1.36 mm^−^^1^
*c* = 19.3011 (4) Å	*T* = 293 (2) K
β = 94.661 (1)º	Prism, colourless
*V* = 3047.82 (12) Å^3^	0.30 × 0.30 × 0.24 mm
*Z* = 4	

### Data collection

**Table:**

Bruker APEXII diffractometer	8699 independent reflections
Radiation source: fine-focus sealed tube	5453 reflections with *I* > 2σ(*I*)
Monochromator: graphite	*R*_int_ = 0.035
*T* = 293(2) K	θ_max_ = 29.8º
ω and φ scans	θ_min_ = 1.7º
Absorption correction: multi-scan(Blessing, 1995)	*h* = −20→19
*T*_min_ = 0.671, *T*_max_ = 0.720	*k* = −15→15
37171 measured reflections	*l* = −26→26

### Refinement

**Table:**

Refinement on *F*^2^	Hydrogen site location: inferred from neighbouring sites
Least-squares matrix: full	H-atom parameters constrained
*R*[*F*^2^ > 2σ(*F*^2^)] = 0.053	*w* = 1/[σ^2^(*F*_o_^2^) + (0.09*P*)^2^ + 1.6651*P*] where *P* = (*F*_o_^2^ + 2*F*_c_^2^)/3
*wR*(*F*^2^) = 0.176	(Δ/σ)_max_ = 0.001
*S* = 1.02	Δρ_max_ = 0.72 e Å^−^^3^
8699 reflections	Δρ_min_ = −0.86 e Å^−^^3^
398 parameters	Extinction correction: SHELXL97 (Sheldrick, 2008), Fc^*^=kFc[1+0.001xFc^2^λ^3^/sin(2θ)]^-1/4^
Primary atom site location: structure-invariant direct methods	Extinction coefficient: 0.0037 (6)
Secondary atom site location: difference Fourier map	

### Fractional atomic coordinates and isotropic or equivalent isotropic displacement parameters (Å^2^)

**Table:**

	*x*	*y*	*z*	*U*_iso_*/*U*_eq_	
C1	0.70233 (19)	0.1671 (3)	0.69974 (13)	0.0414 (6)	
C2	0.68196 (19)	0.0826 (2)	0.75936 (13)	0.0419 (6)	
H2	0.6303	0.0261	0.7486	0.050*	
C3	0.68566 (18)	0.1382 (3)	0.83223 (12)	0.0416 (6)	
H3	0.7007	0.2254	0.8307	0.050*	
C4	0.83576 (18)	0.0507 (2)	0.82017 (12)	0.0383 (5)	
H4	0.8742	−0.0205	0.8336	0.046*	
C5	0.77879 (19)	0.0276 (2)	0.75107 (12)	0.0405 (5)	
H5	0.7777	−0.0580	0.7361	0.049*	
C6	0.85780 (18)	0.1320 (2)	0.64929 (12)	0.0398 (6)	
C7	0.9257 (2)	0.0452 (3)	0.64583 (14)	0.0480 (6)	
H7	0.9233	−0.0258	0.6721	0.058*	
C8	0.9979 (2)	0.0618 (3)	0.60390 (15)	0.0534 (7)	
H8	1.0437	0.0025	0.6021	0.064*	
C9	1.0015 (2)	0.1668 (3)	0.56475 (15)	0.0520 (7)	
C10	0.9323 (2)	0.2523 (3)	0.56705 (16)	0.0556 (7)	
H10	0.9337	0.3220	0.5396	0.067*	
C11	0.8610 (2)	0.2368 (3)	0.60915 (15)	0.0487 (6)	
H11	0.8152	0.2962	0.6107	0.058*	
C12	1.1467 (3)	0.1143 (5)	0.5225 (2)	0.0798 (11)	
H12A	1.1883	0.1430	0.4897	0.120*	
H12B	1.1266	0.0331	0.5100	0.120*	
H12C	1.1781	0.1135	0.5682	0.120*	
C13	0.89041 (17)	0.1991 (2)	0.90386 (12)	0.0330 (5)	
C14	0.87399 (17)	0.3350 (2)	0.91654 (13)	0.0371 (5)	
C15	0.89318 (17)	0.3797 (2)	0.98876 (13)	0.0371 (5)	
C16	0.8704 (2)	0.5000 (3)	1.00480 (17)	0.0519 (7)	
H16	0.8441	0.5516	0.9704	0.062*	
C17	0.8873 (3)	0.5414 (3)	1.07230 (19)	0.0643 (9)	
H17	0.8713	0.6209	1.0833	0.077*	
C18	0.9270 (2)	0.4672 (3)	1.12289 (17)	0.0623 (9)	
H18	0.9381	0.4966	1.1680	0.075*	
C19	0.9505 (2)	0.3509 (3)	1.10802 (14)	0.0495 (7)	
H19	0.9783	0.3013	1.1429	0.059*	
C20	0.93320 (17)	0.3050 (2)	1.04094 (13)	0.0389 (5)	
C21	0.9594 (2)	0.1759 (3)	1.02692 (14)	0.0494 (7)	
H21A	0.9114	0.1219	1.0413	0.059*	
H21B	1.0165	0.1563	1.0545	0.059*	
C22	0.9726 (2)	0.1536 (2)	0.95051 (13)	0.0425 (6)	
H22A	1.0283	0.1950	0.9384	0.051*	
H22B	0.9807	0.0668	0.9429	0.051*	
C23	0.89336 (16)	0.1679 (2)	0.82651 (12)	0.0337 (5)	
H23	0.8584	0.2313	0.7999	0.040*	
C24	0.98856 (18)	0.1624 (3)	0.80023 (13)	0.0444 (6)	
C25	1.0212 (2)	0.2641 (3)	0.76751 (15)	0.0563 (8)	
H25	0.9843	0.3337	0.7630	0.068*	
C26	1.1072 (3)	0.2648 (5)	0.74137 (19)	0.0852 (14)	
H26	1.1281	0.3345	0.7199	0.102*	
C27	1.1604 (3)	0.1645 (6)	0.74713 (19)	0.0958 (18)	
C28	1.1314 (3)	0.0618 (6)	0.7789 (2)	0.0966 (17)	
H28	1.1695	−0.0068	0.7827	0.116*	
C29	1.0447 (3)	0.0594 (4)	0.80593 (18)	0.0716 (10)	
H29	1.0247	−0.0106	0.8275	0.086*	
C30	0.5995 (2)	0.1179 (4)	0.87015 (15)	0.0565 (8)	
H30A	0.6075	0.1552	0.9158	0.068*	
H30B	0.5898	0.0310	0.8762	0.068*	
C31	0.4002 (2)	0.1351 (3)	0.87652 (14)	0.0495 (7)	
C32	0.3946 (3)	0.0177 (4)	0.9000 (2)	0.0728 (10)	
H32	0.4378	−0.0398	0.8878	0.087*	
C33	0.3252 (3)	−0.0167 (4)	0.9417 (2)	0.0842 (12)	
H33	0.3226	−0.0970	0.9572	0.101*	
C34	0.2633 (3)	0.0623 (5)	0.9598 (2)	0.0851 (13)	
H34	0.2168	0.0375	0.9873	0.102*	
C35	0.2670 (3)	0.1785 (5)	0.9386 (2)	0.0879 (14)	
H35	0.2237	0.2344	0.9525	0.106*	
C36	0.3345 (2)	0.2166 (4)	0.8964 (2)	0.0695 (10)	
H36	0.3356	0.2973	0.8815	0.083*	
N1	0.78743 (16)	0.1158 (2)	0.69470 (10)	0.0394 (5)	
N2	0.76374 (16)	0.0684 (2)	0.86869 (10)	0.0419 (5)	
O1	0.80695 (13)	0.13881 (18)	0.92556 (9)	0.0450 (4)	
O2	0.84345 (18)	0.4012 (2)	0.87024 (11)	0.0628 (6)	
O3	1.06894 (17)	0.1926 (3)	0.52152 (14)	0.0757 (8)	
O4	0.66189 (15)	0.2495 (2)	0.66906 (11)	0.0575 (5)	
Cl1	1.26648 (8)	0.1614 (3)	0.71137 (7)	0.1700 (10)	
Se1	0.49162 (2)	0.18861 (4)	0.817879 (17)	0.06640 (16)	

### Atomic displacement parameters (Å^2^)

**Table:**

	*U*^11^	*U*^22^	*U*^33^	*U*^12^	*U*^13^	*U*^23^
C1	0.0462 (14)	0.0452 (15)	0.0319 (11)	−0.0076 (12)	−0.0014 (10)	−0.0051 (10)
C2	0.0448 (14)	0.0466 (15)	0.0340 (11)	−0.0163 (11)	0.0019 (10)	−0.0054 (10)
C3	0.0449 (14)	0.0480 (15)	0.0320 (11)	−0.0129 (12)	0.0034 (10)	−0.0024 (10)
C4	0.0470 (14)	0.0354 (12)	0.0322 (11)	−0.0041 (11)	0.0013 (10)	0.0024 (9)
C5	0.0547 (15)	0.0331 (12)	0.0335 (11)	−0.0091 (11)	0.0020 (10)	−0.0037 (9)
C6	0.0450 (14)	0.0462 (15)	0.0277 (11)	−0.0069 (11)	−0.0004 (9)	−0.0052 (10)
C7	0.0613 (17)	0.0437 (15)	0.0395 (13)	0.0033 (13)	0.0065 (12)	0.0004 (11)
C8	0.0556 (17)	0.0597 (18)	0.0456 (15)	0.0111 (14)	0.0082 (12)	−0.0044 (13)
C9	0.0452 (15)	0.069 (2)	0.0419 (14)	−0.0051 (14)	0.0051 (11)	0.0015 (13)
C10	0.0513 (17)	0.0624 (19)	0.0534 (16)	−0.0034 (15)	0.0055 (13)	0.0174 (15)
C11	0.0461 (15)	0.0539 (17)	0.0461 (14)	0.0023 (13)	0.0037 (11)	0.0076 (13)
C12	0.0520 (19)	0.116 (3)	0.073 (2)	0.006 (2)	0.0159 (17)	−0.004 (2)
C13	0.0358 (11)	0.0332 (12)	0.0295 (10)	−0.0023 (9)	−0.0003 (8)	0.0025 (9)
C14	0.0349 (12)	0.0385 (13)	0.0370 (12)	0.0038 (10)	−0.0020 (9)	0.0025 (10)
C15	0.0344 (12)	0.0364 (13)	0.0407 (12)	−0.0044 (10)	0.0051 (9)	−0.0012 (10)
C16	0.0573 (17)	0.0390 (15)	0.0604 (17)	0.0009 (13)	0.0109 (14)	−0.0014 (13)
C17	0.076 (2)	0.0462 (17)	0.072 (2)	−0.0042 (16)	0.0178 (18)	−0.0197 (16)
C18	0.069 (2)	0.069 (2)	0.0504 (17)	−0.0174 (17)	0.0099 (15)	−0.0185 (15)
C19	0.0516 (16)	0.0585 (18)	0.0381 (13)	−0.0102 (13)	0.0011 (11)	−0.0052 (12)
C20	0.0362 (12)	0.0446 (14)	0.0353 (12)	−0.0061 (10)	−0.0003 (9)	−0.0025 (10)
C21	0.0636 (18)	0.0473 (16)	0.0347 (12)	0.0094 (13)	−0.0108 (12)	0.0033 (11)
C22	0.0509 (15)	0.0379 (13)	0.0371 (12)	0.0117 (11)	−0.0074 (11)	0.0010 (10)
C23	0.0351 (11)	0.0353 (12)	0.0303 (10)	−0.0016 (9)	0.0003 (9)	0.0023 (9)
C24	0.0381 (13)	0.0622 (17)	0.0325 (11)	−0.0017 (12)	−0.0002 (10)	−0.0039 (11)
C25	0.0518 (17)	0.074 (2)	0.0443 (15)	−0.0194 (15)	0.0074 (12)	−0.0075 (14)
C26	0.053 (2)	0.152 (4)	0.0524 (19)	−0.036 (3)	0.0117 (16)	−0.008 (2)
C27	0.0392 (18)	0.209 (6)	0.0390 (17)	0.001 (3)	0.0041 (13)	−0.014 (3)
C28	0.063 (2)	0.169 (5)	0.057 (2)	0.057 (3)	0.0006 (18)	−0.007 (3)
C29	0.065 (2)	0.098 (3)	0.0516 (18)	0.032 (2)	0.0041 (15)	0.0042 (18)
C30	0.0450 (15)	0.086 (2)	0.0392 (14)	−0.0098 (15)	0.0051 (11)	0.0036 (14)
C31	0.0432 (14)	0.0645 (19)	0.0400 (13)	−0.0064 (13)	−0.0017 (11)	0.0043 (12)
C32	0.070 (2)	0.064 (2)	0.086 (3)	0.0009 (18)	0.0116 (19)	0.0029 (19)
C33	0.087 (3)	0.077 (3)	0.087 (3)	−0.023 (2)	0.004 (2)	0.027 (2)
C34	0.051 (2)	0.128 (4)	0.077 (3)	−0.011 (2)	0.0054 (17)	0.030 (3)
C35	0.050 (2)	0.125 (4)	0.090 (3)	0.015 (2)	0.0159 (19)	0.022 (3)
C36	0.0545 (19)	0.075 (2)	0.079 (2)	0.0053 (17)	0.0061 (17)	0.0215 (19)
N1	0.0482 (12)	0.0390 (11)	0.0307 (9)	−0.0051 (9)	0.0012 (8)	−0.0010 (8)
N2	0.0487 (12)	0.0452 (12)	0.0316 (10)	−0.0131 (10)	0.0020 (8)	−0.0011 (9)
O1	0.0508 (11)	0.0568 (11)	0.0272 (8)	−0.0179 (9)	0.0031 (7)	0.0000 (7)
O2	0.0903 (17)	0.0478 (12)	0.0468 (11)	0.0209 (11)	−0.0156 (11)	0.0064 (9)
O3	0.0550 (14)	0.102 (2)	0.0736 (16)	0.0021 (13)	0.0258 (12)	0.0183 (14)
O4	0.0554 (12)	0.0655 (14)	0.0514 (11)	0.0061 (11)	0.0031 (9)	0.0103 (11)
Cl1	0.0433 (5)	0.398 (3)	0.0709 (7)	0.0147 (10)	0.0161 (5)	−0.0191 (12)
Se1	0.0518 (2)	0.0977 (3)	0.0502 (2)	−0.00495 (17)	0.00749 (14)	0.01801 (17)

### Geometric parameters (Å, °)

**Table:**

C1—O4	1.204 (3)	C17—C18	1.361 (5)
C1—N1	1.364 (4)	C17—H17	0.9300
C1—C2	1.523 (4)	C18—C19	1.355 (5)
C2—C3	1.529 (3)	C18—H18	0.9300
C2—C5	1.546 (4)	C19—C20	1.392 (4)
C2—H2	0.9800	C19—H19	0.9300
C3—N2	1.492 (4)	C20—C21	1.494 (4)
C3—C30	1.513 (4)	C21—C22	1.522 (4)
C3—H3	0.9800	C21—H21A	0.9700
C4—N2	1.469 (3)	C21—H21B	0.9700
C4—C23	1.529 (3)	C22—H22A	0.9700
C4—C5	1.531 (3)	C22—H22B	0.9700
C4—H4	0.9800	C23—C24	1.507 (4)
C5—N1	1.468 (3)	C23—H23	0.9800
C5—H5	0.9800	C24—C25	1.382 (4)
C6—C7	1.373 (4)	C24—C29	1.389 (5)
C6—C11	1.388 (4)	C25—C26	1.380 (5)
C6—N1	1.408 (3)	C25—H25	0.9300
C7—C8	1.384 (4)	C26—C27	1.341 (8)
C7—H7	0.9300	C26—H26	0.9300
C8—C9	1.379 (4)	C27—C28	1.363 (7)
C8—H8	0.9300	C27—Cl1	1.734 (4)
C9—O3	1.364 (4)	C28—C29	1.398 (6)
C9—C10	1.374 (5)	C28—H28	0.9300
C10—C11	1.375 (4)	C29—H29	0.9300
C10—H10	0.9300	C30—Se1	1.949 (3)
C11—H11	0.9300	C30—H30A	0.9700
C12—O3	1.414 (5)	C30—H30B	0.9700
C12—H12A	0.9600	C31—C32	1.368 (5)
C12—H12B	0.9600	C31—C36	1.381 (5)
C12—H12C	0.9600	C31—Se1	1.902 (3)
C13—O1	1.467 (3)	C32—C33	1.389 (6)
C13—C22	1.516 (3)	C32—H32	0.9300
C13—C14	1.529 (3)	C33—C34	1.313 (7)
C13—C23	1.536 (3)	C33—H33	0.9300
C14—O2	1.206 (3)	C34—C35	1.339 (6)
C14—C15	1.482 (3)	C34—H34	0.9300
C15—C20	1.387 (4)	C35—C36	1.386 (6)
C15—C16	1.399 (4)	C35—H35	0.9300
C16—C17	1.382 (5)	C36—H36	0.9300
C16—H16	0.9300	N2—O1	1.442 (3)
			
O4—C1—N1	132.7 (3)	C20—C19—H19	119.8
O4—C1—C2	135.2 (3)	C15—C20—C19	119.7 (3)
N1—C1—C2	92.0 (2)	C15—C20—C21	121.6 (2)
C1—C2—C3	117.3 (2)	C19—C20—C21	118.7 (2)
C1—C2—C5	85.76 (19)	C20—C21—C22	112.4 (2)
C3—C2—C5	106.6 (2)	C20—C21—H21A	109.1
C1—C2—H2	114.5	C22—C21—H21A	109.1
C3—C2—H2	114.5	C20—C21—H21B	109.1
C5—C2—H2	114.5	C22—C21—H21B	109.1
N2—C3—C30	108.6 (2)	H21A—C21—H21B	107.9
N2—C3—C2	101.5 (2)	C13—C22—C21	111.6 (2)
C30—C3—C2	114.7 (2)	C13—C22—H22A	109.3
N2—C3—H3	110.6	C21—C22—H22A	109.3
C30—C3—H3	110.6	C13—C22—H22B	109.3
C2—C3—H3	110.6	C21—C22—H22B	109.3
N2—C4—C23	104.4 (2)	H22A—C22—H22B	108.0
N2—C4—C5	102.6 (2)	C24—C23—C4	116.5 (2)
C23—C4—C5	117.4 (2)	C24—C23—C13	115.8 (2)
N2—C4—H4	110.6	C4—C23—C13	101.93 (18)
C23—C4—H4	110.6	C24—C23—H23	107.4
C5—C4—H4	110.6	C4—C23—H23	107.4
N1—C5—C4	117.7 (2)	C13—C23—H23	107.4
N1—C5—C2	87.3 (2)	C25—C24—C29	118.1 (3)
C4—C5—C2	105.9 (2)	C25—C24—C23	118.5 (3)
N1—C5—H5	114.2	C29—C24—C23	123.4 (3)
C4—C5—H5	114.2	C26—C25—C24	121.5 (4)
C2—C5—H5	114.2	C26—C25—H25	119.2
C7—C6—C11	119.3 (3)	C24—C25—H25	119.2
C7—C6—N1	120.0 (2)	C27—C26—C25	119.6 (5)
C11—C6—N1	120.7 (2)	C27—C26—H26	120.2
C6—C7—C8	121.0 (3)	C25—C26—H26	120.2
C6—C7—H7	119.5	C26—C27—C28	121.2 (4)
C8—C7—H7	119.5	C26—C27—Cl1	120.3 (5)
C9—C8—C7	119.6 (3)	C28—C27—Cl1	118.5 (4)
C9—C8—H8	120.2	C27—C28—C29	120.1 (4)
C7—C8—H8	120.2	C27—C28—H28	120.0
O3—C9—C10	116.0 (3)	C29—C28—H28	120.0
O3—C9—C8	124.6 (3)	C24—C29—C28	119.5 (4)
C10—C9—C8	119.4 (3)	C24—C29—H29	120.2
C9—C10—C11	121.3 (3)	C28—C29—H29	120.2
C9—C10—H10	119.4	C3—C30—Se1	110.4 (2)
C11—C10—H10	119.4	C3—C30—H30A	109.6
C10—C11—C6	119.5 (3)	Se1—C30—H30A	109.6
C10—C11—H11	120.2	C3—C30—H30B	109.6
C6—C11—H11	120.2	Se1—C30—H30B	109.6
O3—C12—H12A	109.5	H30A—C30—H30B	108.1
O3—C12—H12B	109.5	C32—C31—C36	117.1 (3)
H12A—C12—H12B	109.5	C32—C31—Se1	123.3 (3)
O3—C12—H12C	109.5	C36—C31—Se1	119.6 (3)
H12A—C12—H12C	109.5	C31—C32—C33	120.7 (4)
H12B—C12—H12C	109.5	C31—C32—H32	119.6
O1—C13—C22	107.84 (19)	C33—C32—H32	119.6
O1—C13—C14	104.49 (19)	C34—C33—C32	121.1 (4)
C22—C13—C14	110.4 (2)	C34—C33—H33	119.4
O1—C13—C23	105.42 (18)	C32—C33—H33	119.4
C22—C13—C23	114.9 (2)	C33—C34—C35	120.0 (4)
C14—C13—C23	112.90 (19)	C33—C34—H34	120.0
O2—C14—C15	121.9 (2)	C35—C34—H34	120.0
O2—C14—C13	121.2 (2)	C34—C35—C36	120.9 (4)
C15—C14—C13	116.9 (2)	C34—C35—H35	119.6
C20—C15—C16	119.1 (2)	C36—C35—H35	119.6
C20—C15—C14	121.6 (2)	C31—C36—C35	120.2 (4)
C16—C15—C14	119.3 (2)	C31—C36—H36	119.9
C17—C16—C15	119.4 (3)	C35—C36—H36	119.9
C17—C16—H16	120.3	C1—N1—C6	134.1 (2)
C15—C16—H16	120.3	C1—N1—C5	94.9 (2)
C18—C17—C16	120.9 (3)	C6—N1—C5	130.8 (2)
C18—C17—H17	119.6	O1—N2—C4	105.64 (18)
C16—C17—H17	119.6	O1—N2—C3	110.5 (2)
C19—C18—C17	120.5 (3)	C4—N2—C3	108.43 (19)
C19—C18—H18	119.8	N2—O1—C13	109.84 (17)
C17—C18—H18	119.8	C9—O3—C12	118.3 (3)
C18—C19—C20	120.5 (3)	C31—Se1—C30	97.81 (12)
C18—C19—H19	119.8		
			
O4—C1—C2—C3	−72.1 (4)	C14—C13—C23—C24	95.7 (3)
N1—C1—C2—C3	106.3 (3)	O1—C13—C23—C4	−23.4 (2)
O4—C1—C2—C5	−178.7 (3)	C22—C13—C23—C4	95.2 (2)
N1—C1—C2—C5	−0.26 (18)	C14—C13—C23—C4	−136.9 (2)
C1—C2—C3—N2	−116.6 (2)	C4—C23—C24—C25	143.3 (2)
C5—C2—C3—N2	−22.8 (2)	C13—C23—C24—C25	−96.9 (3)
C1—C2—C3—C30	126.5 (3)	C4—C23—C24—C29	−35.4 (4)
C5—C2—C3—C30	−139.6 (3)	C13—C23—C24—C29	84.4 (3)
N2—C4—C5—N1	117.0 (2)	C29—C24—C25—C26	−0.4 (4)
C23—C4—C5—N1	3.3 (4)	C23—C24—C25—C26	−179.1 (3)
N2—C4—C5—C2	21.6 (2)	C24—C25—C26—C27	0.6 (5)
C23—C4—C5—C2	−92.1 (3)	C25—C26—C27—C28	−0.6 (6)
C1—C2—C5—N1	0.25 (17)	C25—C26—C27—Cl1	177.2 (3)
C3—C2—C5—N1	−117.0 (2)	C26—C27—C28—C29	0.4 (6)
C1—C2—C5—C4	118.3 (2)	Cl1—C27—C28—C29	−177.5 (3)
C3—C2—C5—C4	1.0 (3)	C25—C24—C29—C28	0.2 (5)
C11—C6—C7—C8	1.1 (4)	C23—C24—C29—C28	178.9 (3)
N1—C6—C7—C8	−177.1 (3)	C27—C28—C29—C24	−0.2 (6)
C6—C7—C8—C9	−0.3 (5)	N2—C3—C30—Se1	−172.35 (18)
C7—C8—C9—O3	−179.8 (3)	C2—C3—C30—Se1	−59.6 (3)
C7—C8—C9—C10	−1.2 (5)	C36—C31—C32—C33	0.3 (6)
O3—C9—C10—C11	−179.4 (3)	Se1—C31—C32—C33	−178.8 (3)
C8—C9—C10—C11	1.9 (5)	C31—C32—C33—C34	0.0 (7)
C9—C10—C11—C6	−1.1 (5)	C32—C33—C34—C35	−0.8 (7)
C7—C6—C11—C10	−0.4 (4)	C33—C34—C35—C36	1.4 (7)
N1—C6—C11—C10	177.7 (3)	C32—C31—C36—C35	0.3 (5)
O1—C13—C14—O2	−96.4 (3)	Se1—C31—C36—C35	179.4 (3)
C22—C13—C14—O2	147.9 (3)	C34—C35—C36—C31	−1.1 (7)
C23—C13—C14—O2	17.7 (4)	O4—C1—N1—C6	−5.6 (5)
O1—C13—C14—C15	80.9 (2)	C2—C1—N1—C6	176.0 (3)
C22—C13—C14—C15	−34.9 (3)	O4—C1—N1—C5	178.7 (3)
C23—C13—C14—C15	−165.1 (2)	C2—C1—N1—C5	0.28 (19)
O2—C14—C15—C20	−176.3 (3)	C7—C6—N1—C1	−162.9 (3)
C13—C14—C15—C20	6.5 (3)	C11—C6—N1—C1	19.0 (4)
O2—C14—C15—C16	3.8 (4)	C7—C6—N1—C5	11.4 (4)
C13—C14—C15—C16	−173.4 (2)	C11—C6—N1—C5	−166.7 (2)
C20—C15—C16—C17	−0.7 (4)	C4—C5—N1—C1	−106.8 (3)
C14—C15—C16—C17	179.3 (3)	C2—C5—N1—C1	−0.28 (19)
C15—C16—C17—C18	1.0 (5)	C4—C5—N1—C6	77.3 (3)
C16—C17—C18—C19	−0.3 (5)	C2—C5—N1—C6	−176.2 (2)
C17—C18—C19—C20	−0.8 (5)	C23—C4—N2—O1	−33.7 (2)
C16—C15—C20—C19	−0.4 (4)	C5—C4—N2—O1	−156.62 (19)
C14—C15—C20—C19	179.7 (2)	C23—C4—N2—C3	84.8 (2)
C16—C15—C20—C21	−179.8 (3)	C5—C4—N2—C3	−38.1 (2)
C14—C15—C20—C21	0.3 (4)	C30—C3—N2—O1	−84.9 (3)
C18—C19—C20—C15	1.1 (4)	C2—C3—N2—O1	153.83 (18)
C18—C19—C20—C21	−179.5 (3)	C30—C3—N2—C4	159.7 (2)
C15—C20—C21—C22	22.0 (4)	C2—C3—N2—C4	38.5 (2)
C19—C20—C21—C22	−157.4 (3)	C4—N2—O1—C13	18.9 (2)
O1—C13—C22—C21	−56.5 (3)	C3—N2—O1—C13	−98.2 (2)
C14—C13—C22—C21	57.1 (3)	C22—C13—O1—N2	−119.7 (2)
C23—C13—C22—C21	−173.8 (2)	C14—C13—O1—N2	122.8 (2)
C20—C21—C22—C13	−50.9 (3)	C23—C13—O1—N2	3.6 (2)
N2—C4—C23—C24	161.9 (2)	C10—C9—O3—C12	173.6 (3)
C5—C4—C23—C24	−85.5 (3)	C8—C9—O3—C12	−7.8 (5)
N2—C4—C23—C13	34.9 (2)	C32—C31—Se1—C30	−48.0 (3)
C5—C4—C23—C13	147.6 (2)	C36—C31—Se1—C30	132.9 (3)
O1—C13—C23—C24	−150.9 (2)	C3—C30—Se1—C31	175.9 (2)
C22—C13—C23—C24	−32.3 (3)		

### Hydrogen-bond geometry (Å, °)

**Table:**

*D*—H···*A*	*D*—H	H···*A*	*D*···*A*	*D*—H···*A*
C4—H4···O4^i^	0.98	2.57	3.305 (3)	132
C23—H23···O2	0.98	2.32	2.803 (3)	109
C23—H23···N1	0.98	2.54	2.918 (3)	103

Symmetry codes: (i) −*x*+3/2, *y*−1/2, −*z*+3/2.
